# Appropriate regulation of routine laboratory testing can reduce the costs associated with patient stay in intensive care

**DOI:** 10.1186/cc9553

**Published:** 2011-03-11

**Authors:** K Goddard, SJ Austin

**Affiliations:** 1 Mater Hospital, Belfast, UK

## Introduction

Traditionally within our ICU, comprehensive daily bloods were taken on a routine basis without direct clinician involvement. Such routine blood testing can be costly [1], time consuming, labour intensive, and can contribute to patient anaemia [2]. Recognising these concerns, a new clinician-centred system for ordering blood tests was implemented in July 2010. This was based on a blood investigation order chart completed by medical personnel to specify the blood tests required for individual patients for the following day. The objective of this audit was therefore to assess whether the implementation of the blood investigation order chart reduced the number of blood tests performed and the associated costs.

## Methods

Data on the numbers and types of blood investigations were collated for all patients with a length of stay greater than 24 hours in our six-bed critical care unit. The audit period covered 100 days prior to implementation of the order chart and 100 days post implementation. The blood tests assessed were; full blood picture (FBP), urea and electrolytes (U&E), coagulation screen, liver function tests (LFT), magnesium, bone profile (Ca, PO_4 _and albumin), and C-reactive protein (CRP). A comparative analysis of the numbers, types and costs of blood testing pre and post implementation was conducted. The study did not seek to assess patient outcomes mainly due to the small number of patients involved.

## Results

The implementation of the ordering chart resulted in a reduction in the number of blood investigations ordered, from a total of 2,209 pre implementation to 1,477 post implementation; that is, a 33% net reduction. The tests that showed the largest reductions were coagulation screens, LFT and bone profiles, with reductions of 52%, 54% and 53%, respectively. A moderate reduction was observed in magnesium and CRP tests, at 43% and 21% respectively. Only a very small reduction in the number of FBP and U&E tests was found. When the financial costs of these reductions are assessed, the analysis showed an overall saving for the ICU of £17,914 per annum, or £2,986 per bed.

## Conclusions

The results of this audit suggests that the implementation of simple low-cost measures, such as a blood investigation order chart to specify and customise blood testing in the ICU, can significantly reduce the costs associated with patient stay in the ICU.
